# Occupational Class and Cancer Survival in Korean Men: Follow-Up Study of Nation-Wide Working Population

**DOI:** 10.3390/ijerph17010303

**Published:** 2020-01-01

**Authors:** Hye-Eun Lee, Masayoshi Zaitsu, Eun-A Kim, Ichiro Kawachi

**Affiliations:** 1 Korea Institute of Labor Safety and Health, Seoul 07023, Korea; heunn.lee@gmail.com; 2 Department of Social and Behavioral Sciences, Harvard T.H. Chan School of Public Health, Boston, MA 02115, USA; m-zaitsu@m.u-tokyo.ac.jp (M.Z.); ikawachi@hsph.harvard.edu (I.K.); 3 Department of Public Health, Graduate School of Medicine, The University of Tokyo, Tokyo 113-0033, Japan; 4 Occupational Safety and Health Research Institute, Korea Occupational Safety and Health Agency, Ulsan 44429, Korea

**Keywords:** socioeconomic factors, occupations, survival, Korea

## Abstract

*Background*: We aimed to describe inequalities in site-specific cancer survival across different occupational classes in Korean men. *Methods*: Subjects included cancer diagnosed members of the national employment insurance program during 1995–2008. A total of 134,384 male cases were followed by linking their data to the Death registry until 2009. Occupational classes were categorized according to the Korean Standard Occupational Classification (KSOC). Hazard ratio adjusting age and diagnosed year were calculated for each occupation by cancer sites. *Results*: Men in service/sales and blue-collar occupations had lower survival of all cancer sites combined and esophagus, stomach, colorectal, liver, larynx, lung, prostate, thyroid cancer and non-Hodgkin’s lymphoma than men in professional and managerial positions. Cancer sites with good prognosis like prostate cancer showed wider gap across occupational class. *Conclusions*: Considerable inequalities in cancer survival were found by occupation among Korean men. Cancer control policy should more focus on lower socioeconomic occupational class.

## 1. Introduction

Improving cancer survival is a key element of cancer control. However, socioeconomic disparities in cancer survival have been documented, even in countries which have universal health care systems, such as the UK or Canada [1,2]. In a review of 42 studies on social class disparities in cancer survival based in North America and western European countries, wider gaps were found for cancer sites with good prognosis, such as female breast, corpus uteri, bladder and colon cancer [3]. The possible reasons for these disparities include delayed detection, variations in the quality of cancer treatment, as well as health behaviors (including therapeutic compliance) [4].

Although extensive studies have documented socioeconomic disparities in cancer survival in Western settings, studies in the Asian context remain sparse [5,6]. In South Korea, previous studies have been published on socioeconomic inequalities in cancer survival, but these have been limited to single cancer sites [7,8] and/or unrepresentative samples [9,10]. In these previous studies, the indicators to assess socioeconomic status (SES) included educational level, income (health insurance premium as a surrogate for income), medical insurance status and area-level socioeconomic indices. Although occupation is widely used in research of health inequalities [11], there have been no previous studies focusing on occupational disparities in cancer survival in Korea.

Therefore, the aim of this study was to examine cancer survival disparities according to occupational class using a nationwide sample. In this study, we focus on male cancer disparities due to the ample sample size for classifying occupations.

## 2. Materials and Methods

### 2.1. Data Source and Study Population

Our data comprises a cohort of Korean workers assembled from the national Employment Insurance database (1995–2000). Workers nationwide employed in the private sector have been required to join the employment insurance program in Korea under the Employment Insurance Act since 1995. The database included 11,435,937 employed workers in Korea. We excluded foreigners, workers under the age of <15 years or >60 years (statutory age of retirement) at baseline, as well as workers with inaccurate enrollment dates. We restricted the cohort to men, and also excluded men with missing occupational data or who changed occupation during 1995–2000.

Patients with a diagnosis of malignant neoplasm (C00–C97) according to the International Classification of Disease, 10th Revision (ICD-10), were confirmed by matching to the Korea Central Cancer Registry (KCCR) (1995–2008). Data from 2008 was the latest available cancer registry data at the time of data collection. ICD-10 codes for cancer diagnoses were provided by the KCCR, which originally classified cancer cases according to International Classification of Diseases for Oncology 3rd edition and converted into ICD-10 codes [12].

**Figure 1:**
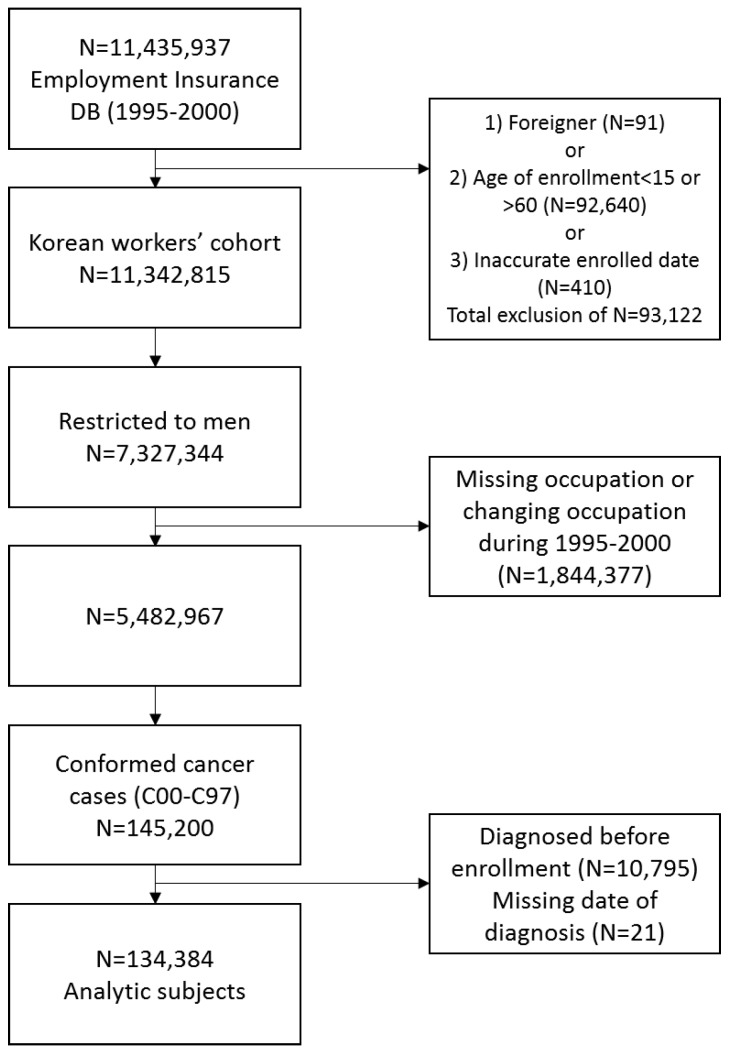
Selection of study population.

Individuals were excluded if they were diagnosed with cancer before enrollment or had missing information on date of diagnosis. These exclusions resulted in a study population of 134,384 male cancer cases (Figure 1). In the present study, recurrent cancer cases were not included, but second primary cancers among the same individuals were considered.

### 2.2. Calculation of Survival

The date and cause of death was confirmed by matching our data with the death registry maintained by the Korea National Statistical Office (KNSO) between 1995 and 2009. Mortality data up to the year of 2009 were the latest available data, although we tried to follow the individual as long as possible to ensure enough endpoints. The average follow-up period (from date of diagnosis to date of death or end of study) was 3.2 years.

Cancer-specific survival was calculated from the date of cancer diagnosis to date of death from cancer. Death from non-cancer cause or undefined cause were not counted as “endpoint” and censored at the date of death. Since 4.5% of causes of death were undefined, for sensitivity analysis, we also re-ran all the analyses where survival was calculated from the date of cancer diagnosis to the date of death regardless of cause of death (Supplementary Table S1).

### 2.3. Occupational Class

The Korean workers cohort included the date of entering and/or leaving the employment insurance program (surrogate for employment and retirement date). The cohort also included information on industry and occupation (nine categories) coded with the Korean Standard Classification of Occupations (KSCO) between 1995 and 2000. This classification is comparable to the International Standard Classification of Occupations [13]. For comparing across occupational classes with sufficient sample sizes, we collapsed the nine occupational classes into four SES groups as follows: Group 1 (Professionals and managers) included KSCO1 (legislators, senior officials and managers), KSCO2 (professionals) and KSCO3 (technicians and associate professionals); Group 2 (Clerical) included KSCO4 (clerks); Group 3 (Service and sales workers) included KSCO5 (service workers and sale workers); Group 4 (Blue-collar workers) included KSCO6 (agricultural, forestry, and fishery workers), KSCO7 (craft and related trades workers), KSCO8 (plant and machine operators, and assemblers) and KSCO9 (elementary occupations) [13].

### 2.4. Statistical Analysis

To compare cancer survival across occupational class, Cox proportional hazards models were used to calculate hazard ratios (HRs) and 95% confidence intervals (95% CIs), adjusting for age and year of diagnosis as continuous variables, because age at diagnosis and year of diagnosis were both significant determinants of cancer survival in univariate analysis (Supplementary Table S2). Analyses were performed for all cancer sites (C00–C97) and specific cancer sites that had sufficient number of cases (more than 1000). A two-side *p* value < 0.05 was regarded statistically significant. All analyses were performed using the SAS software (version 9.4; SAS Inc., Cary, NC, USA).

### 2.5. Ethics Statement

This study was approved by the institutional review boards of the Occupational Safety and Health Research Institute and Korea Occupational Safety and Health Agency, Ulsan, Korea.

## 3. Results

In total, 134,384 cancer cases were available for analysis (Table 1). Around 60% of the sample were aged more than 50 years when they were diagnosed with cancer. Group 4 (blue-collar workers) was the most numerous occupational class. During the follow-up period, 64,267 men died.

The distribution of cancer by type is presented in Table 2. Common cancer sites were stomach (24.2%), liver (17.2%), colon and rectum (13.7%), lung (10.7%) and thyroid gland cancer (4.5%).

Table 3 shows hazard ratio (HR) and 95% confidence intervals (CIs) of cancer specific survival with Group 1 (professionals and managers) as the reference group. Lower SES occupations (Service/sales workers (Group 3) and Blue-collar workers (Group 4)) showed poorer survival for all cancer sites combined as well as many specific cancer sites. The magnitude of the differences was wider (HR higher than 1.5) for cancers with good prognosis, including lip, oral cavity, pharynx (C00–C14), larynx (C32), prostate (C61), kidney (C64) and thyroid gland (C73).

**Figure 2:**
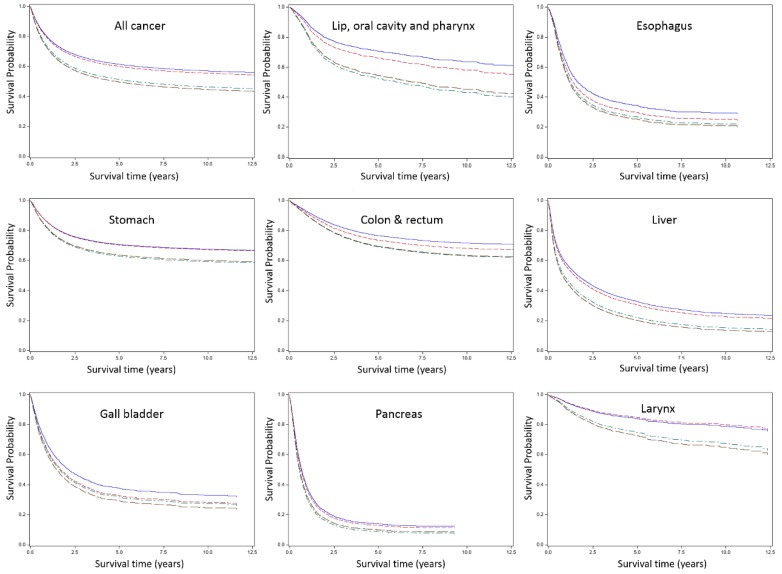

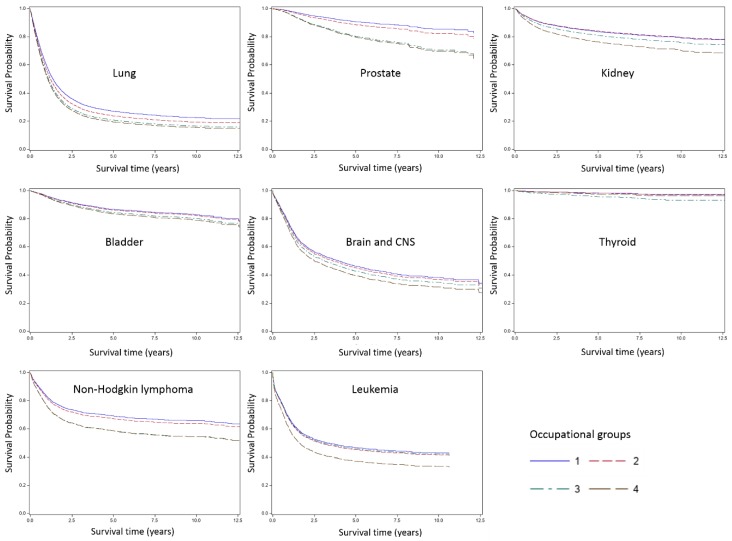
Survival curve by occupational group and cancer site. Group 1, Professionals and managers; Group 2, Clerks; Group 3, Service and sales workers; Group 4, Blue-collar workers.

Figure 2 presents survival curves for all cancer and specific cancer sites by occupational groups. Although the differences across occupational groups varied, higher SES occupational group showed better survival for most cancer sites. Generally, cancer sites with poor prognosis (e.g., pancreas and lung) showed narrower survival differences, while cancer sites with good prognosis (e.g., larynx and prostate) tended to show wider disparities. In the case of thyroid cancer, lower SES occupational group showed high HR (2.6 for service and sales workers and 1.4 for blue-collar workers), but the difference in survival curves across groups were not noteworthy compared to the other cancer sites, because survival for thyroid cancer was extremely good for almost all participants.

The results of cox-proportional model for overall survival is presented in Table S1. There was no considerable difference between the HRs of overall survival and cancer specific survival.

## 4. Discussion

We found significant disparities in cancer survival by occupation among Korean working men. The disparities were more pronounced for cancer sites that are generally considered to have good prognosis, suggesting that variations in stage of diagnosis or treatment quality may have contributed to these patterns. We found the widest gap between occupational classes for survival following prostate cancer and thyroid cancer diagnosis (they are the least fatal cancer in Korean males with higher than 90% 5-year survival rate [14]). However, survival from thyroid cancer was extremely good in general, so that the absolute differences between occupational class was not clearly discernible in survival curves, though the relative difference was large. In the case of pancreas cancer which has the lowest 5-year survival (less than 10%) in the general population, the occupational class gap was the narrowest (although still statistically significant).

Stage at diagnosis is the most well established factor contributing to socioeconomic disparity in cancer survival [15]. In Korea, the National Cancer Screening Program (NCSP) currently provides free access to cancer screening for stomach, colorectal, liver, female breast and cervical cancer (there is a 10% of copayment for individuals with high income) [16]. Although lack of income is not a barrier for receiving screening, nevertheless, it has been shown that low income groups are less likely to participate in the national screening program [16]. As a result of the low participation rates, individuals in the bottom quartile of income have been shown to have a roughly 1.3 times higher risk of being diagnosed with stomach and colorectal cancer at an advanced stage [17]. Besides the national screening program, private cancer screening with extra payment is widely available throughout Korea. Many clinics and hospitals offer private cancer screening as a “package” to paying customers, and these packages usually include more items than the NCSP, for example, tumor markers such as prostate-specific antigen (PSA), colonoscopy (as opposed to fecal occult blood testing under the national program) and sonography (for abdomen, thyroid and breast). For more extensive packages, a low-dose Computed Tomography (CT) of chest or abdomen is often added. Due to the extra cost, access to private cancer screening is more affordable for higher income groups. According to a previous study, male office workers showed higher participation rates than male manual workers in private cancer screening [18].

At the same time, not every form of cancer screening is medically appropriate. For example, thyroid and prostate cancer are widely cited as instances of excessive screening and overdiagnosis [19]. In the present study, thyroid and prostate cancer survival showed the widest gap between occupational classes. However, in the previous study with same data source on cancer incidence difference across occupational class, higher occupational class men (managers and professionals) showed significantly higher age-standardized incidence of thyroid, kidney and prostate cancer [20].

Besides disparities in screening and detection, another major explanation for survival disparities is differences in treatment quality or intensity [21,22]. In Korea, there is universal access to healthcare under the National Health Insurance (NHI) or Medicaid program. In a previous study, including around 1000 patients with stomach, colon, liver and lung cancer, there was no difference in the number of outpatient visit per month comparing high versus low income groups [10]. Nevertheless, under the NHI, patients still have to pay 5% of the cost of cancer treatment out-of-pocket. Besides financial reasons, knowledge (health literacy), time constraints and other available resources could affect health care utilization by lower SES groups.

A third explanation for occupational disparities in cancer survival is driven by health behaviors. Health behaviors are generally considered to be plausible mediators of social inequalities in health, as they are strongly socially patterned and concurrently associated with health outcomes [23]. For example, smoking status both before and after lung cancer diagnosis is an important factor for survival [24,25]. Similarly, alcohol could affect survival following liver cancer diagnosis [26]. Therefore, behavior is one potential mediator of occupational class disparities in cancer survival, although we could not evaluate contribution of behavioral factors due to lack of information.

Lastly, psychosocial factors including social support may affect cancer survival. For example, the survival advantage of having a partner for cancer patients is well known and a married male could benefit more than females [27,28]. The need for social support was shown to be inversely associated with income level among Korean cancer patients [29]. Insufficient social support could be associated with cancer survival via delayed diagnosis or improper choice of treatment [21].

Currently, the most commonly diagnosed cancer sites among Korean men are stomach, lung and colorectal cancer [30]. However, our data showed a higher number of liver cancers than lung or colorectal cancer, which reflects the high rates of liver cancer during the time period of our study. Until about 1999, liver cancer mortality was higher than lung cancer mortality in Korea, although the incidence of liver cancer has been declining since. The major risk factors for liver cancer in Korea are hepatitis B virus infection and alcoholic liver cirrhosis [31].

Our study has some strengths. First, due to linkage between a large and representative workers cohort and cancer registry data, we identified almost all cancer cases among employed workers in the private sector in Korea during study period. Sufficient cases were available to evaluate specific cancer sites; therefore, we could compare the magnitude of association between different cancer sites. To our knowledge, this is the first study to investigate cancer survival across different occupational class using nationwide data in Korea. Second, our longitudinal follow-up design helps to rule out reverse causation, i.e., downward occupational “drift” as a result of cancer diagnosis. Third, information on occupational classification was obtained from the Employment Insurance data, which is provided from the companies employing the individuals; hence, the possibility of misclassification of occupation is expected to be lower than when the information is collected by self-report.

This study also had a number of limitations. We lacked information on stage at diagnosis, pathological subtypes, treatment information or other covariates, including behavior or occupational exposure. Thus, we could not evaluate the impact of mediators between occupational class and cancer survival. Second, there could be nondifferential misclassification of occupation; hence, we could not totally exclude the possibility that the results were underestimated. In addition, occupational information was available during 1995–2000; therefore, we could not reflect possible change of occupational class of cancer patients diagnosed after 2000.

## 5. Conclusions

In conclusion, lower socioeconomic occupational class could be a risk factor for cancer survival among Korean working men. Subgroup analysis by cancer sites showed the associations between occupational class and specific cancer survival, and the magnitude of association was larger in cancer sites with good prognosis. Further investigation is needed to identify the factors mediating occupational class and cancer survival. Service/sales workers and blue-collar workers should be considered as a target population for more intensive cancer control efforts.

## Figures and Tables

**Table 1:** Characteristics of 134,384 male cancer patients.

	N	%
Age of Diagnosis		
<20	42	0.0
20–29	2,156	1.6
30–39	13,590	10.1
40–49	31,780	23.7
50–59	46,729	34.8
60–	40,087	29.8
Year of diagnosis		
1995–1999	19,804	14.7
2000–2004	49,771	37.0
2005–2008	64,820	48.2
Occupational group		
Group 1 (Professionals and managers)	28,198	21.0
Group 2 (Clerks)	23,937	17.8
Group 3 (Service and sales workers)	8,152	6.1
Group 4 (blue-collar workers)	74,097	55.1
Vital status		
Alive	70,117	52.2
Dead	64,267	47.8
cause of death: cancer	58,332	(90.8)
cause of death: non-cancer	3,037	(4.7)
cause of death: undefined	2,898	(4.5)
Total	134,384	100.0

**Table 2:** Distribution of cancer sites in the study population.

Site of Cancer	N	%
All cancer (C00–C97)	134,384	100.0
Lip, oral cavity and pharynx (C00–C14)	3142	2.3
Esophagus (C15)	2192	1.6
Stomach (C16)	32,524	24.2
Colon, rectosigmoid junction, rectum (C18–C20)	18,361	13.7
Liver and intrahepatic bile ducts (C22)	23,170	17.2
Gallbladder, other and unspecified parts of biliary tract (C23–C24)	2632	2.0
Pancreas (C25)	3068	2.3
Larynx (C32)	1496	1.1
Trachea, bronchus and lung (C33–C34)	14,423	10.7
Mesothelioma (C45)	92	0.1
Breast (C50)	102	0.1
Prostate (C61)	3403	2.5
Testis (C62)	443	0.3
Kidney (C64)	3746	2.8
Bladder (C67)	3329	2.5
Brain and other parts of central nervous system (C70–C72)	1775	1.3
Thyroid gland (C73)	6059	4.5
Hodgkin lymphoma (C81)	264	0.2
Non-Hodgkin lymphoma (C82–C85, C96)	3706	2.8
Multiple myeloma (C90)	701	0.5
Leukemia (C91–C95)	2748	2.0

Group 1, Professionals and managers; Group 2, Clerks; Group 3, Service and sales workers; Group 4, Blue-collar workers.

**Table 3:** Cancer specific HRs and 95% CIs according to occupational groups using Cox proportional hazard model adjusted for age and year of diagnosis.

		Adjusted HR	95% CI
All cancer (C00–C97)	Group 1	Reference	
	Group 2	1.05	1.02–1.08
	Group 3	1.38	1.33–1.43
	Group 4	1.45	1.42–1.48
Lip, oral cavity and pharynx (C00-C14)	Group 1	Reference	
	Group 2	1.20	0.97–1.49
	Group 3	1.89	1.47–2.42
	Group 4	1.78	1.51–2.10
Esophagus (C15)	Group 1	Reference	
	Group 2	1.14	0.91–1.41
	Group 3	1.24	0.94–1.61
	Group 4	1.29	1.11–1.51
Stomach (C16)	Group 1	Reference	
	Group 2	1.01	0.95–1.09
	Group 3	1.35	1.23–1.47
	Group 4	1.31	1.24–1.38
Colon, rectosigmoid junction, rectum (C18–C20)	Group 1	Reference	
	Group 2	1.15	1.05–1.27
	Group 3	1.39	1.22–1.57
	Group 4	1.37	1.27–1.47
Liver and intrahepatic bile ducts (C22)	Group 1	Reference	
	Group 2	1.06	1.01–1.12
	Group 3	1.36	1.26–1.46
	Group 4	1.44	1.38–1.50
Gallbladder, other and unspecified parts of biliary tract (C23–C24)	Group 1	Reference	
	Group 2	1.14	0.96–1.36
	Group 3	1.18	0.93–1.47
	Group 4	1.26	1.11–1.44
Pancreas (C25)	Group 1	Reference	
	Group 2	1.04	0.91–1.19
	Group 3	1.23	1.03–1.46
	Group 4	1.18	1.06–1.30
Larynx (C32)	Group 1	Reference	
	Group 2	0.94	0.57–1.55
	Group 3	1.66	0.96–2.78
	Group 4	1.84	1.33–2.60
Trachea, bronchus and lung (C33–C34)	Group 1	Reference	
	Group 2	1.10	1.02–1.19
	Group 3	1.21	1.10–1.33
	Group 4	1.26	1.19–1.33
Prostate (C61)	Group 1	Reference	
	Group 2	1.25	0.89–1.74
	Group 3	2.26	1.39–3.52
	Group 4	2.35	1.85–3.02
Kidney (C64)	Group 1	Reference	
	Group 2	0.98	0.77–1.24
	Group 3	1.19	0.84–1.65
	Group 4	1.53	1.28–1.85
Bladder (C67)	Group 1	Reference	
	Group 2	1.04	0.76–1.42
	Group 3	1.18	0.75–1.81
	Group 4	1.26	1.01–1.60
Brain and other parts of central nervous system (C70–C72)	Group 1	Reference	
	Group 2	1.04	0.85–1.27
	Group 3	1.11	0.84–1.45
	Group 4	1.22	1.04–1.45
Thyroid gland (C73)	Group 1	Reference	
	Group 2	1.07	0.63–1.79
	Group 3	2.59	1.25–4.98
	Group 4	1.43	0.94–2.20
Non-Hodgkin lymphoma (C82–C85,C96)	Group 1	Reference	
	Group 2	1.08	0.90–1.29
	Group 3	1.46	1.15–1.84
	Group 4	1.46	1.27–1.68
Leukemia (C91–C95)	Group 1	Reference	
	Group 2	1.04	0.89–1.23
	Group 3	1.03	0.82–1.27
	Group 4	1.32	1.16–1.50

## Supplementary Materials

The following are available online at https://www.mdpi.com/1660-4601/17/1/303/s1, Table S1: Overall HRs and their 95% CIs according to occupational groups using Cox proportional hazard model. adjusted for age and year of diagnosis, Table S2: Results of univariate analysis of cancer-specific survival for all cancer combined.
