# Supplementary material for: Occupational Class and Cancer Survival in Korean Men: Follow-Up Study of Nation-Wide Working Population
Source: Int J Environ Res Public Health. 2020 Jan 1;17(1):303. doi: 10.3390/ijerph17010303 (PMC6981645; doi:10.3390/ijerph17010303)
Supplement: Supplementary file 1 [file ijerph-17-00303-s001.pdf]

Supplementary

Table S1. Overall HRs and their 95% CIs according to occupational groups using Cox proportional hazard model adjusted for age and year of diagnosis.

		Adjusted HR	95% CI
All cancer(C00-C97)	Group 1	Reference	
	Group 2	1.06	1.03 - 1.09
	Group 3	1.39	1.34 - 1.44
	Group 4	1.48	1.45 - 1.51
Lip, oral cavity and pharynx (C00-C14)	Group 1	Reference	
	Group 2	1.18	0.97 - 1.45
	Group 3	1.78	1.39 - 2.25
	Group 4	1.79	1.54 - 2.10
Esophagus (C15)	Group 1	Reference	
	Group 2	1.20	0.97 - 1.48
	Group 3	1.32	1.02 - 1.70
	Group 4	1.33	1.15 - 1.55
Stomach (C16)	Group 1	Reference	
	Group 2	1.03	0.97 - 1.10
	Group 3	1.36	1.25 - 1.49
	Group 4	1.36	1.29 - 1.43
Colon, rectosigmoid junction, rectum (C18-C20)	Group 1	Reference	
	Group 2	1.14	1.04 - 1.25
	Group 3	1.44	1.28 - 1.63
	Group 4	1.43	1.33 - 1.54
Liver and intrahepatic bile ducts (C22)	Group 1	Reference	
	Group 2	1.07	1.01 - 1.12
	Group 3	1.36	1.27 - 1.45
	Group 4	1.46	1.40 - 1.52
Gallbladder, other and unspecified parts of biliary tract (C23-C24)	Group 1	Reference	
	Group 2	1.17	0.99 - 1.38
	Group 3	1.16	0.93 - 1.45
	Group 4	1.28	1.13 - 1.45
Pancreas (C25)	Group 1	Reference	
	Group 2	1.08	0.95 - 1.23
	Group 3	1.25	1.05 - 1.48
	Group 4	1.20	1.09 - 1.33
Larynx (C32)	Group 1	Reference	

	Group 2	1.04	0.65 - 1.63
	Group 3	1.49	0.87 - 2.46
	Group 4	1.96	1.45 - 2.72
Trachea, bronchus and lung (C33-C34)	Group 1	Reference	
	Group 2	1.11	1.04 - 1.20
	Group 3	1.22	1.11 - 1.33
	Group 4	1.27	1.20 - 1.34
Prostate (C61)	Group 1	Reference	
	Group 2	1.35	0.99 - 1.83
	Group 3	1.97	1.22 - 3.04
	Group 4	2.32	1.85 - 2.93
Kidney (C64)	Group 1	Reference	
	Group 2	0.99	0.79 - 1.23
	Group 3	1.28	0.93 - 1.72
	Group 4	1.58	1.34 - 1.88
Bladder (C67)	Group 1	Reference	
	Group 2	1.15	0.87 - 1.52
	Group 3	1.28	0.85 - 1.88
	Group 4	1.36	1.11 - 1.69
Brain and other parts of central nervous system (C70-C72)	Group 1	Reference	
	Group 2	1.02	0.85 - 1.24
	Group 3	1.05	0.81 - 1.35
	Group 4	1.20	1.03 - 1.40
Thyroid gland (C73)	Group 1	Reference	
	Group 2	1.01	0.64 - 1.59
	Group 3	2.23	1.15 - 4.03
	Group 4	1.54	1.08 - 2.25
Non-Hodgkin lymphoma (C82-C85,C96)	Group 1	Reference	
	Group 2	1.07	0.90 - 1.26
	Group 3	1.46	1.17 - 1.80
	Group 4	1.47	1.29 - 1.68
Leukemia (C91-C95)	Group 1	Reference	
	Group 2	1.03	0.88 - 1.20
	Group 3	1.03	0.83 - 1.26
	Group 4	1.34	1.18 - 1.52

Group 1, Professional and manager; Group 2, Clerks; Group 3, Service and sales workers; Group 4, Blue-collar workers.

Table S2. Results of univariate analysis of cancer-specific survival for all cancer combined.

		Hazard Ratio	95% Confidence Interval
Age at diagnosis	15-19	1.40	0.86 – 2.13
	20-29	1.15	1.06 – 1.23
	30-39	Reference	
	40-49	1.33	1.29 – 1.38
	50-59	1.68	1.63 – 1.74
	60-69	1.76	1.70 – 1.82
Year of diagnosis	2005-2008	Reference	
	2000-2004	1.34	1.32 – 1.37
	1995-1999	1.65	1.61 – 1.69
